# Conservative Surgical Treatment of a Case of Placenta Accreta

**DOI:** 10.1055/s-0038-1668528

**Published:** 2018-08

**Authors:** Ismail Biyik, Fatih Keskin, Elif Usturali Keskin

**Affiliations:** 1 Department of Obstetrics and Gynecology, Karacabey State Hospital, Bursa, Turkey; 2 Department of Obstetrics and Gynecology, Mustafakemalpasa State Hospital, Bursa, Turkey; 3 Department of Medical Pathology, Mustafakemalpasa State Hospital, Bursa, Turkey

**Keywords:** placenta accreta, cesarean delivery, hysterectomy prevention

## Abstract

Placenta accreta syndromes are associated with increased maternal mortality and morbidity. Cesarean hysterectomy is usually performed in cases of placenta accreta syndrome. Fertility sparing methods can be applied. In the present study, we report a successful segmental uterine resection method for placenta accreta in the anterior uterine wall in a cesarean section case. A 39-year-old woman underwent an elective cesarean section at 38 + 2 weeks. A placental tissue with an area of 10 cm was observed extending from the anterior uterine wall to the serosa, 2 cm above the uterine incision line. The placental tissue was removed with the help of monopolar electrocautery. The uterine incision was continuously sutured. The patient was discharged on the second postoperative day. The placental pathology was reported as placenta accreta. The American College of Obstetricians and Gynecologists (ACOG) generally recommends cesarean section hysterectomy in cases of placenta accreta because removal of placenta associated with significant hemorrhage. Conservative and fertility sparing methods include placenta left in situ, cervical inversion technique and triple-P procedure. There are several studies reporting that segmental uterine resection is performed with and without balloon placement or artery ligation. Segmental uterine resection may be an alternative to cesarean hysterectomy to preserve fertility or to protect the uterus in cases of placenta accreta when there is no placenta previa.

## Introduction

Normal placentation occurs as a result of the placement of the placenta in the decidua. The result of placenta adherence to the myometrium instead of to the decidua results in placenta accreta. Abnormal adhesion is named according to the extent of myometrium and uterine serosal involvement. All of these abnormalities are called placenta accreta syndrome. The most common type of placenta invasion anomaly is placenta accreta, and the most serious is placenta percreta, which is related with the increase in cesarean delivery rates. The incidence of placenta invasion anomalies is up to 1/533 pregnancies.^1^ Placenta accreta syndromes are associated with increased maternal mortality and morbidity.^2^ Cesarean hysterectomy is usually performed in cases of placenta accreta syndrome. Nowadays, fertility sparing and conservative methods can be applied. These methods include placenta left in situ, cervical inversion technique and triple-P procedure.^3^ ^4^ Placenta left in situ and methotrexate use have serious risks, such as late postpartum hemorrhage, infection, and pulmonary embolism. In the cervical inversion technique, the cervix is inverted using ring forceps or straight Allis forceps, after which the placental bed is sutured to control bleeding.^4^ In the triple-P procedure, a balloon is placed preoperatively in the hypogastric arteries and the balloon is inflated after the baby is born. Recently, a limited number of cases of segmental uterine resection have been reported. We reported a successful segmental uterine resection method for placenta accreta in the anterior uterine wall in a cesarean section case.

## Case Report

**Fig. 1:**
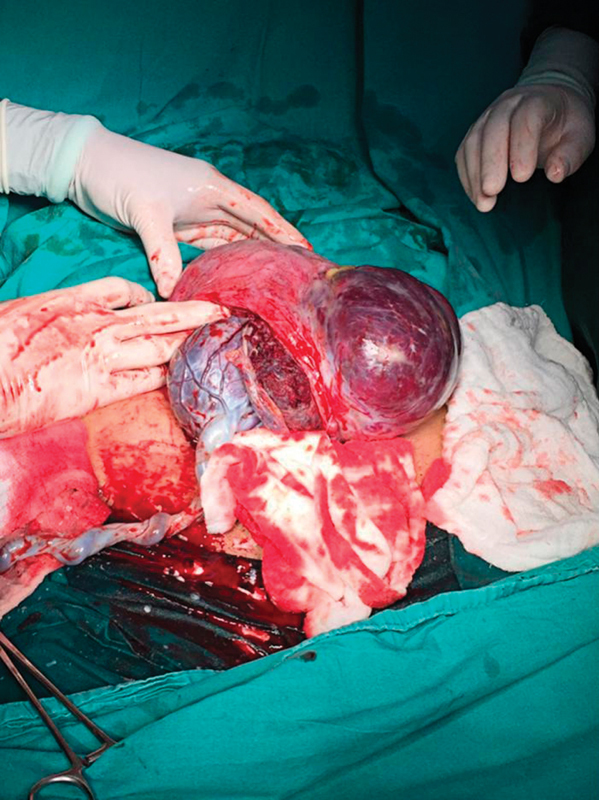
Placental tissue was observed ∼ 2 cm above the uterine incision line.

**Fig. 2:**
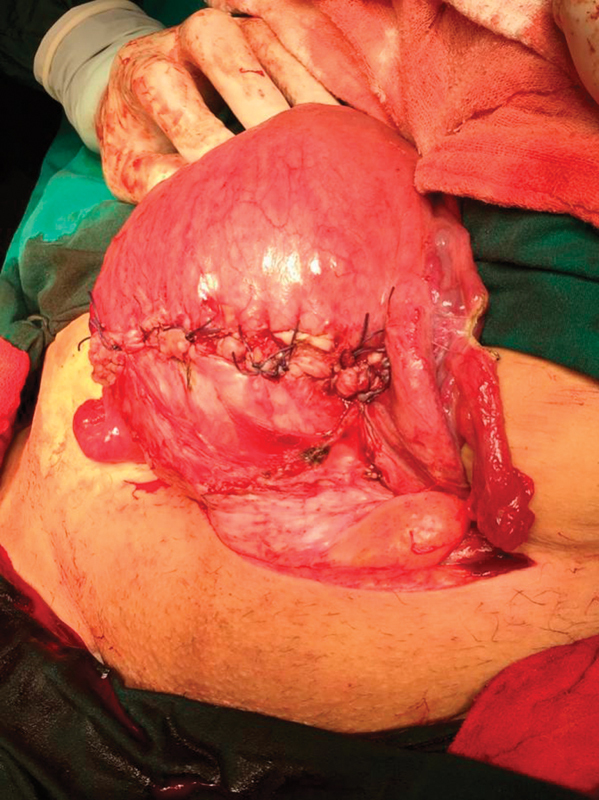
Reconstruction of the uterine wall.

A 39-year-old, gravida 4, para 3 pregnant woman underwent an elective cesarean section at 38 + 2 weeks. The patient had a history of two previous cesarean sections. Under regional anesthesia, the cesarean section was performed with Pfannenstiel incision and transverse uterine incision. The patient had no placenta accreta diagnosis preoperatively. A healthy 3,100 g male newborn was delivered. Twenty IU of oxytocin (Synptian Fort, Deva, Turkey) was intravenously administered after the delivery of the fetus and the removal of the involved area. The placental tissue was observed extending from the anterior uterine wall to the serosa ∼ 2 cm above the uterine incision line (Fig. 1). Placenta percreta was thought with intraoperative. The placenta was not removed due to the possibility of bleeding. The area of 10 cm, which is considered to be a placenta percreta, was removed with the help of monopolar electrocautery. The remaining placenta fragments were removed with gentle traction. The uterine incision was continuously sutured with no.1 vicryl (Polyglactin 910 suture, Doğsan, Trabzon, Turkey) (Fig. 2). Hypogastric or uterine artery ligation was not performed because there was no intensive bleeding. Due to the preoperative approval, tubal ligation was performed with the Pomeroy method. The estimated amount of bleeding was not calculated. A total of 3,000 mL of crystalloids and 500 mL of colloid fluid were administered intraoperatively, assuming that the amount of bleeding was of 1,000 mL. Immediately, 3 units of erythrocyte suspension were prepared for transfusion. The operation was completed in 60 minutes. Hemoglobin 10.3 g/dL, hematocrit value of 31.4% in the preoperative period; hemoglobin 8.5 g/dL and hematocrit value of 31.4% after the transfusion of 1 unit of erythrocyte suspension in the postoperative period. Intraoperative and postoperative complications did not develop. The patient was discharged on the second postoperative day. The placental pathology was reported as a placenta accreta.

## Discussion

Obstetric hemorrhage due to placenta accreta syndrome is one of the important reasons of maternal mortality and morbidity. The American College of Obstetricano and Gynecologists (ACOG) generally recommends cesarean section hysterectomy in cases of placenta accreta because removal of placenta associated with significant hemorrhage.^5^ However, conservative and fertility sparing methods can be applied in selected cases.^6^ Subsequently, the uterine wall containing the placenta accreta is removed.^3^ Another method is to perform bilateral hypogastric artery ligation intraoperatively, after the removal of the baby, and to perform segmental uterine resection of the placenta percreta area.^7^ ^8^ There are some studies reporting that segmental uterine resection is performed without balloon placement or artery ligation.^9^ The duration of the operation is increased in cases of placenta previa with uterine artery ligation.^7^ The duration of the operation is shortened when arterial ligation and balloon placement are not performed.^9^ Due to the absence of placenta previa and the absence of arterial ligation in our case, the operation was completed within 60 minutes. The amount of bleeding due to the absence of placenta previa was lower than that reported in other studies. For this reason, 1 unit of erythrocyte suspension was sufficient.

## Conclusion

Cesarean hysterectomy is usually performed in cases of placenta accreta syndrome. Segmental uterine resection may be an alternative to cesarean hysterectomy, to preserve fertility or to protect the uterus, in cases in which there is no placenta previa

## References

[1] Wu S Kocherginsky M Hibbard J U Abnormal placentation: twenty-year analysis Am J Obstet Gynecol 2005 192 05 1458 1461 Doi: 10.1016/j.ajog.2004.12.074 15902137 10.1016/j.ajog.2004.12.074

[2] Chan B C Lam H S Yuen J H Conservative management of placenta praevia with accreta Hong Kong Med J 2008 14 06 479 484 19060348

[3] Chandraharan E Rao S Belli A M Arulkumaran S The Triple-P procedure as a conservative surgical alternative to peripartum hysterectomy for placenta percreta Int J Gynaecol Obstet 2012 117 02 191 194 Doi: 10.1016/j.ijgo.2011.12.005 22326782 10.1016/j.ijgo.2011.12.005

[4] Sakhavar N Heidari Z Mahmoudzadeh-Sagheb H Cervical inversion as a novel technique for postpartum hemorrhage management during cesarean delivery for placenta previa accreta/increta Int J Gynaecol Obstet 2015 128 02 122 125 Doi: 10.1016/j.ijgo.2014.08.020 25467911 10.1016/j.ijgo.2014.08.020

[5] Committee on Obstetric Practice. Committee opinion no. 529: placenta accreta Obstet Gynecol 2012 120 01 207 211 Doi: 10.1097/AOG.0b013e318262e340 22914422 10.1097/AOG.0b013e318262e340

[6] Lin K Qin J Xu K Hu W Lin J Methotrexate management for placenta accreta: a prospective study Arch Gynecol Obstet 2015 291 06 1259 1264 Doi: 10.1007/s00404-014-3573-1 25501835 10.1007/s00404-014-3573-1

[7] Karaman E Kolusarı A Çetin O Local resection may be a strong alternative to cesarean hysterectomy in conservative surgical management of placenta percreta: experiences from a tertiary hospital J Matern Fetal Neonatal Med 2017 30 08 947 952 Doi: 10.1080/14767058.2016.1192119 27268514 10.1080/14767058.2016.1192119

[8] Kilicci C Sanverdi I Ozkaya E Segmental resection of anterior uterine wall in cases with placenta percreta: a modified technique for fertility preserving approach J Matern Fetal Neonatal Med 2018 31 09 1198 1203 Doi: 10.1080/14767058.2017.1311862 28349762 10.1080/14767058.2017.1311862

[9] Polat I Yücel B Gedikbasi A Aslan H Fendal A The effectiveness of double incision technique in uterus preserving surgery for placenta percreta BMC Pregnancy Childbirth 2017 17 01 129 Doi: 10.1186/s12884-017-1262-3 28449642 10.1186/s12884-017-1262-3 PMC5406983

